# Mortality trends, sex, and racial disparities in older adults due to abdominal aortic aneurysm: a nationwide cross-sectional analysis

**DOI:** 10.1097/JS9.0000000000002114

**Published:** 2024-10-21

**Authors:** Aman Goyal, Humza Saeed, Syeda Shahnoor, Muhammad Khubaib Arshad, Abdul Wasay, Abdullah, Mohamed Daoud, Amir Humza Sohail

**Affiliations:** a Department of Internal Medicine, Seth GS Medical College and KEM Hospital, Mumbai, India; b Department of Internal Medicine, Rawalpindi Medical University, Rawalpindi, Punjab, Pakistan; c Department of Internal Medicine, Dow University of Health Sciences, Karachi, Pakistan; d Bogomolets National Medical University, Kyiv, Ukraine; e Department of Surgery, University of New Mexico Health Sciences, Albuquerque, NM, USA

**Keywords:** abdominal aortic aneurysm, cardiology, cardiovascular, epidemiology, mortality, racial disparity, sex disparity, trends

## Abstract

Abdominal aortic aneurysms (AAAs) are a significant vascular pathology in older adults, often asymptomatic but with high mortality upon rupture. Despite advancements in diagnostic imaging and surgical interventions, AAAs remain a public health concern. This research letter analyzed CDC WONDER data on AAA-related deaths (ICD-10 I71.3 and I71.4) among US adults aged 65+ from 1999 to 2020. Age-adjusted mortality rates (AAMRs) and annual percent change (APC) were calculated by year, sex, age group, race/ethnicity, geography, and urbanization status. Between 1999 and 2020, there were 180 037 reported deaths in older adults due to AAA. The overall AAMR decreased from 32.6 to 13.2 per 100 000, with a significant decline from 2014 to 2020 (APC: −1.66; 95% CI: −2.48 to −0.48). Older men had a significantly higher AAMR than older women (31.2 vs. 12). Among racial and ethnic groups, Non-Hispanic (NH) Whites had the highest AAMR at 21.7, followed by NH American Indian or Alaska Native (14.5), NH Black (12.6), NH Asian or Pacific Islander (10.1), and Hispanic populations (8.4). Additionally, non-metropolitan areas exhibited higher AAMRs compared to metropolitan areas (23.9 vs. 18.7). While mortality rates have declined, disparities remain, with higher rates among older men, NH Whites, and non-metropolitan residents, highlighting the need for targeted and equitable interventions.

## Graphical abstract

**Figure:**
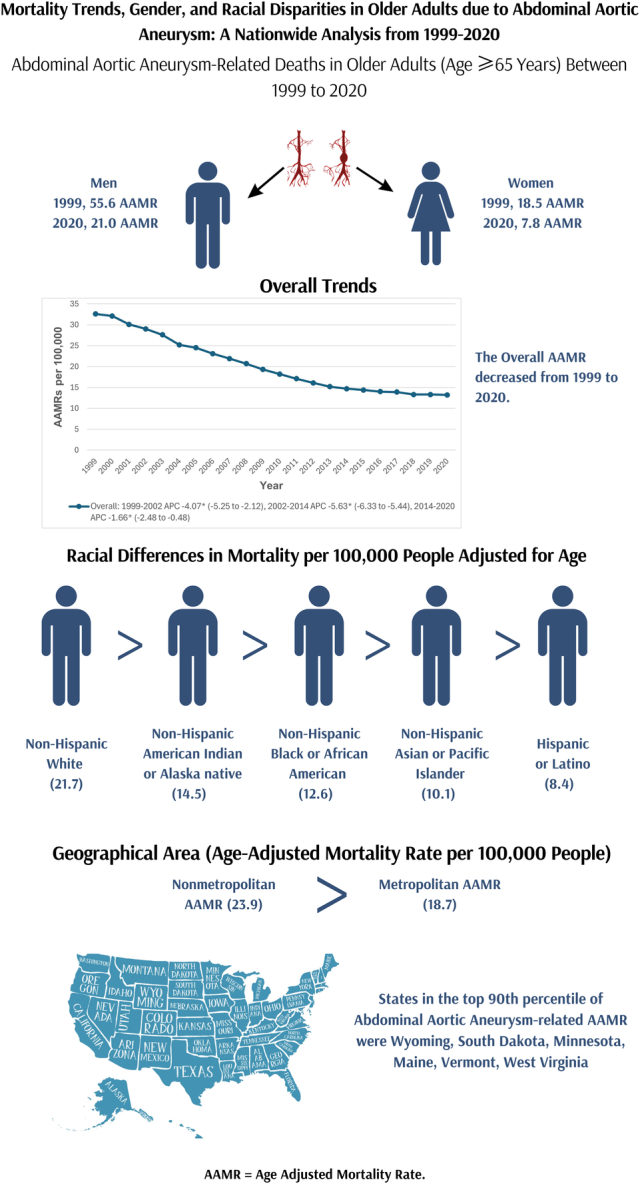

## Introduction

Highlights

- The study found a significant decrease in age-adjusted mortality rates (AAMRs) for abdominal aortic aneurysms (AAAs) among US adults aged 65 and older, dropping from 32.6 to 13.2 per 100 000 population between 1999 and 2020, with a notable decline from 2014 to 2020.
- The study highlights that non-Hispanic Whites have the highest AAMR at 21.7, followed by non-Hispanic American Indian or Alaska Native (14.5), non-Hispanic Black or African American (12.6), non-Hispanic Asian or Pacific Islander (10.1), and Hispanic (8.4) populations, emphasizing the impact of racial and geographic factors on AAA mortality.
- Despite the overall decline in AAA-related mortality, significant disparities remain. Older men, non-Hispanic Whites, and residents of non-metropolitan areas exhibit higher mortality rates, indicating the need for targeted and equitable healthcare interventions.

Abdominal aortic aneurysms (AAAs), defined as a dilation of the abdominal aorta greater than or equal to 3 cm, are a critical vascular condition with a high mortality rate upon rupture, especially among older adults^1^. Survival rates are low, with only half of those reaching the hospital surviving, and overall mortality approaching 90%. Although advancements in diagnostic imaging, surgical techniques, and medical therapies have improved outcomes, AAAs remain a public health concern due to their asymptomatic nature until rupture^1^. Globally, 35 million people are affected, with 170 000 deaths and 3 million disability-adjusted life years reported in 2017^2^.

Screening programs targeting high-risk groups, particularly older men, have reduced mortality, yet disparities in outcomes persist due to socioeconomic, geographic, and healthcare access factors^1^. This study aims to analyze trends and disparities in age-adjusted mortality rates (AAMR) related to AAA among older adults in the United States (US) from 1999 to 2020, focusing on demographic, geographic, and urbanization factors to inform targeted public health interventions.

## Methodology

We analyzed mortality data from the CDC WONDER database, focusing on older adults (65+) who died due to AAAs in the US from 1999 to 2020. AAA-related deaths were identified using ICD-10 codes I71.3 and I71.4 from the Multiple Cause-of-Death Public Use Record database. This database and method have been applied in previously published studies analyzing the mortality data^3^. The data were categorized by demographics, geographic location, urbanization, and place of death. AAMRs were calculated per 100 000 people, standardized to the 2000 US population. Temporal trends in AAMRs were analyzed using Joinpoint regression program (Version 5.0.2, National Cancer Institute), allowing for the identification of up to four inflection points over the 22-year study period. Annual percent change (APC) and 95% CIs were estimated, with statistical significance set at *P* less than or equal to 0.05. The cross-sectional study followed the STROCSS, Supplemental Digital Content 1, http://links.lww.com/JS9/D512 guidelines and did not require ethical approval from the institutional review board approval^4^.

## Results

Between 1999 and 2020, 180 037 deaths among older adults were attributed to AAA, either as an underlying or contributing cause (Supplemental Table 1, Supplemental Digital Content 2, http://links.lww.com/JS9/D513). Of these, the place of death was recorded for 175 186 cases: 65.3% in medical facilities, 19% at home, 11% in nursing homes or long-term care facilities, and 2.2% in hospices (Supplemental Table 2, Supplemental Digital Content 2, http://links.lww.com/JS9/D513).

### Demographic trends in mortality

**Figure 1:**
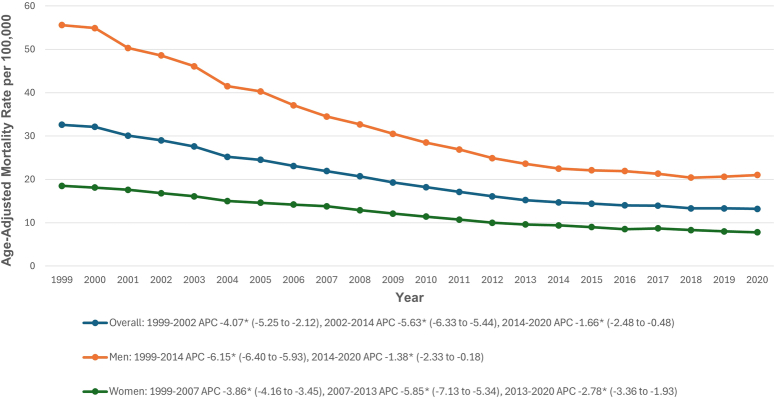
Overall and sex-stratified abdominal aortic aneurysm-related age-adjusted mortality rates per 100 000 in older adults in the United States, 1999–2020. *Indicates that the annual percentage change (APC) is significantly different from zero at α = 0.05.

The overall AAMR for older adults related to AAA was 32.6 in 1999, decreasing to 13.2 in 2020. AAMR significantly decreased from 1999 to 2002 (APC: −4.07; 95% CI: −5.25 to −2.12), from 2002 to 2014 (APC: −5.63; 95% CI: −6.33 to −5.44), and again from 2014 to 2020 (APC: −1.66; 95% CI: −2.48 to −0.48) (Fig. 1, Supplemental Tables 3 and 4, Supplemental Digital Content 2, http://links.lww.com/JS9/D513).

### Sex stratification

Older men had higher AAMRs than older women (31.2 vs. 12). In men, AAMR decreased significantly from 1999 to 2014 (APC: −6.15; 95% CI: −6.40 to −5.93) and continued to decline from 2014 to 2020 (APC: −1.38; 95% CI: −2.33 to −0.18). In women, AAMR decreased from 1999 to 2007 (APC: −3.86; 95% CI: −4.16 to −3.45) and from 2007 to 2013 (APC: −5.85; 95% CI: −7.13 to −5.34), with a continued decline from 2013 to 2020 (APC: −2.78; 95% CI: −3.36 to −1.93) (Fig. 1, Supplemental Tables 3 and 4, Supplemental Digital Content 2, http://links.lww.com/JS9/D513).

### Racial stratification

**Figure 2:**
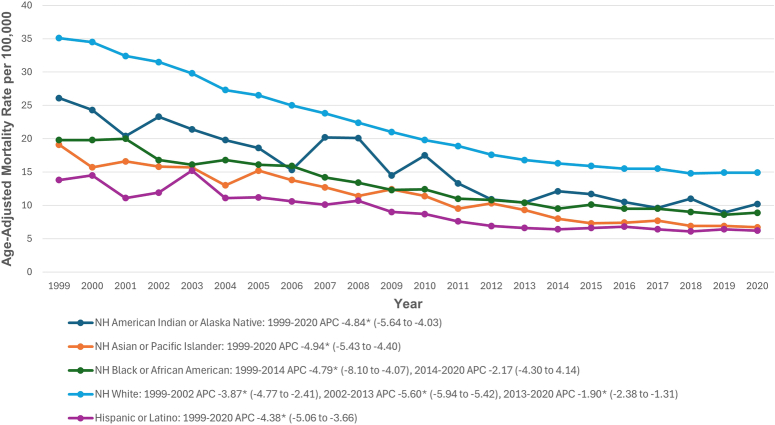
Abdominal aortic aneurysm-related age-adjusted mortality rates per 100 000, stratified by race in older adults in the United States, 1999–2020. *Indicates that the annual percentage change (APC) is significantly different from zero at α = 0.05. NH, non-Hispanic.

AAMRs were highest among non-Hispanic (NH) Whites (21.7), followed by NH American Indian/Alaska Natives (14.5), NH Black/African Americans (12.6), NH Asian/Pacific Islanders (10.1), and Hispanics (8.4). Significant decreases from 1999 to 2020 were observed for NH American Indian/Alaska Natives (APC: −4.84; 95% CI: −5.64 to −4.03), NH Asian/Pacific Islanders (APC: −4.94; 95% CI: −5.43 to −4.40), and Hispanics (APC: −4.38; 95% CI: −5.06 to −3.66). Among NH Black/African Americans, AAMR declined from 1999 to 2014 (APC: −4.79; 95% CI: −8.10 to −4.07) and stabilized from 2014 to 2020. NH Whites experienced significant decreases from 1999 to 2002 (APC: −3.87; 95% CI: −4.77 to −2.41), 2002 to 2013 (APC: −5.60; 95% CI: −5.94 to −5.42), and 2013 to 2020 (APC: −1.90; 95% CI: −2.38 to −1.31) (Fig. 2, Supplemental Tables 3 and 5, Supplemental Digital Content 2, http://links.lww.com/JS9/D513)

### State-wise distribution

**Figure 3:**
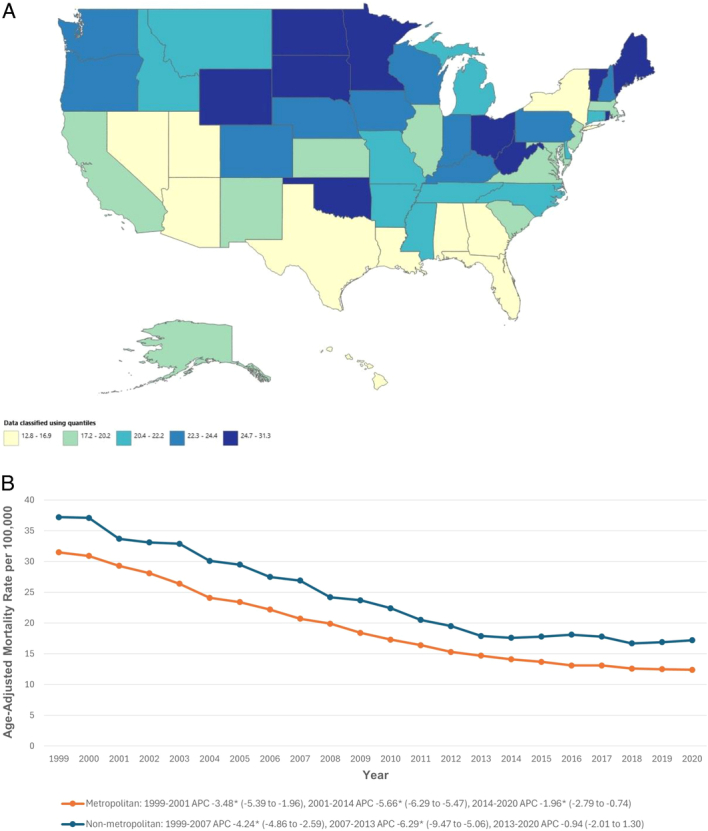
Abdominal aortic aneurysm-related age-adjusted mortality rates per 100 000, stratified by (A) state and (B) urbanization in older adults in the United States, 1999–2020. *Indicates that the annual percentage change (APC) is significantly different from zero at α = 0.05.

AAMRs varied by state, ranging from 12.8 in the District of Columbia to 31.3 in West Virginia. States in the top 90th percentile (e.g. Wyoming, South Dakota, Minnesota, Maine, Vermont, West Virginia) had AAMRs nearly three times higher than those in the bottom 10th percentile (e.g. Hawaii, Alabama, Arizona, Georgia, Utah, District of Columbia) (Fig. 3A, Supplemental Table 6, Supplemental Digital Content 2, http://links.lww.com/JS9/D513).

### Urbanization

Non-metropolitan areas had higher AAMRs than metropolitan areas (23.9 vs. 18.7). In non-metropolitan areas, AAMR significantly decreased from 1999 to 2007 (APC: −4.24; 95% CI: −4.86 to −2.59) and from 2007 to 2013 (APC: −6.29; 95% CI: −9.47 to -5.06), then stabilized from 2013 to 2020. In metropolitan areas, AAMR decreased from 1999 to 2001 (APC: −3.48; 95% CI: −5.39 to −1.96), from 2001 to 2014 (APC: −5.66; 95% CI: −6.29 to −5.47), and again from 2014 to 2020 (APC: −1.96; 95% CI: −2.79 to −0.74) (Fig. 3B, Supplemental Tables 3 and 7, Supplemental Digital Content 2, http://links.lww.com/JS9/D513).

## Discussion

Abdominal aortic aneurysm (AAA) remains a significant health concern, with primary causes of mortality including severe complications such as rupture leading to massive internal bleeding, dissection obstructing blood flow, embolism causing stroke or limb ischemia, and infection progressing to sepsis^1,5,6^. Ruptured AAAs contribute to numerous deaths annually, ranking as the 15th leading cause of death overall and the 10th leading cause among men aged 55 years and older^7^. The key risk factors for AAA mortality include old age, smoking, and family history^1^.

Our study observed a significant reduction in AAA-related mortality rates in older adults, with a 59.6% decrease from 1999 to 2020. This decline aligns with previous statistics and studies from the US, which report a decline in AAA-related mortality^8^. This consistent decline can be attributed to various factors, including primary prevention efforts such as smoking prevention along with healthcare advancements and better screening practices^1,9^. In addition, a major advancement in AAA management is the adoption of endovascular aortic aneurysm repair (EVAR), a minimally invasive technique that has become more popular than open surgical repair due to lower morbidity and faster recovery times. The shift towards EVAR has been associated with decreased AAA-related mortality^10^.

In our study, 65.3% of AAA-related deaths occurred in medical facilities, reflecting the severity of the condition. Older men had nearly 2.5 times higher mortality rates than older women, consistent with studies showing a higher prevalence of AAA in men^1^. Although AAA is less common in women, they face a higher risk of rupture and mortality compared to men with similar-sized aneurysms^11^.

Racial disparities were evident, with the highest mortality rates observed in older NH White individuals, while NH Black, NH Asian, and Hispanic populations had lower rates. This aligns with previous studies depicting a higher prevalence and mortality in white individuals^12,13^. Geographically, significant variations in mortality were observed, with the highest rates in West Virginia and the lowest in the District of Columbia. In terms of urbanization status, non-metropolitan areas had higher mortality rates than metropolitan areas, likely due to limited healthcare access and resources, including timely diagnosis, elective repairs, and specialized interventions, exacerbated by longer travel times to medical facilities and limited resources in rural regions^14^.

Limitations of our study include potential underreporting or misclassification of AAA-related deaths due to reliance on ICD codes and death certificates. In addition, the absence of critical clinical data, including baseline comorbidities and socioeconomic variables, was not accounted for in the dataset, which could lead to potential confounding of the results. Despite these limitations, our analysis highlights the need for targeted public health interventions to address disparities in AAA care and ensure equitable access to prevention and treatment. Continuing to advance both preventive and therapeutic strategies is essential to further decrease the burden of AAA and improve health outcomes for all affected individuals.

## Ethical approval

No ethical approval was required for this study design, as all data were obtained from publicly available sources.

## Consent

Informed consent was not required as we utilized data from a de-identified government-provided public use dataset.

## Source of funding

Not applicable.

## Author contribution

A.A.: study concept and design, data analysis, writing the paper, supervision. H.S.: data analysis or interpretation, methodologies, writing the paper. S.S.: methodologies, data analysis, writing the paper. M.K.A.: data interpretation and analysis, writing the paper. A.W.: methodologies, writing the paper. A.: writing the paper, formatting and editing the final version. M.D.: writing the paper, review of final draft. A.H.S.: study concept and design, writing the paper, review of the final draft, supervision.

## Conflicts of interest disclosure

The authors declare no conflicts of interest.

## Research registration unique identifying number (UIN)

Not applicable.

## Guarantor

Aman Goyal.

## Data availability statement

The data supporting the findings of this study are openly available in CDC Wonder at [https://wonder.cdc.gov/].

## Provenance and peer review

Not commissioned, externally peer-reviewed.

## Supplementary Material

SUPPLEMENTARY MATERIAL
