# Supplementary material for: Mortality trends, sex, and racial disparities in older adults due to abdominal aortic aneurysm: a nationwide cross-sectional analysis
Source: Int J Surg. 2024 Oct 21;110(12):8241–5. doi: 10.1097/JS9.0000000000002114 (PMC11634149; doi:10.1097/JS9.0000000000002114)
Supplement: SUPPLEMENTARY MATERIAL [file js9-110-8241-s002.docx]

**Supplementary File**

**Supplemental Table 1** Abdominal Aortic Aneurysm-related Mortality, Stratified by Sex and Race in older adults in the United States, 1999 to 2020.

| Deaths | | | | | | | | | |
| --- | --- | --- | --- | --- | --- | --- | --- | --- | --- |
| Year | **Overall** | **Women** | **Men** | **NH White** | **NH Black or African American** | **NH Asian or Pacific Islander** | **NH American Indian or Alaska Native** | **Hispanic or Latino** | **Population** |
| 1999 | 11257 | 4005 | 7252 | 10357 | 524 | 127 | 30 | 196 | 34797841 |
| 2000 | 11189 | 3942 | 7247 | 10264 | 534 | 109 | 33 | 221 | 34991753 |
| 2001 | 10625 | 3899 | 6726 | 9725 | 544 | 129 | 25 | 179 | 35290291 |
| 2002 | 10351 | 3747 | 6604 | 9505 | 460 | 126 | 31 | 199 | 35522207 |
| 2003 | 9978 | 3632 | 6346 | 9090 | 442 | 137 | 27 | 260 | 35863529 |
| 2004 | 9216 | 3415 | 5801 | 8382 | 470 | 119 | 26 | 204 | 36203319 |
| 2005 | 9102 | 3358 | 5744 | 8242 | 460 | 148 | 26 | 216 | 36649798 |
| 2006 | 8735 | 3324 | 5411 | 7885 | 463 | 143 | 22 | 212 | 37164107 |
| 2007 | 8405 | 3265 | 5140 | 7597 | 420 | 140 | 31 | 213 | 37825711 |
| 2008 | 8116 | 3114 | 5002 | 7292 | 408 | 134 | 32 | 242 | 38777621 |
| 2009 | 7724 | 2955 | 4769 | 6941 | 386 | 154 | 21 | 215 | 39623175 |
| 2010 | 7364 | 2840 | 4524 | 6573 | 396 | 145 | 30 | 209 | 40267984 |
| 2011 | 7151 | 2726 | 4425 | 6421 | 362 | 132 | 24 | 203 | 41394141 |
| 2012 | 6878 | 2622 | 4256 | 6115 | 373 | 155 | 20 | 194 | 43145356 |
| 2013 | 6724 | 2553 | 4171 | 5973 | 366 | 153 | 22 | 199 | 44704074 |
| 2014 | 6652 | 2544 | 4108 | 5912 | 354 | 139 | 26 | 203 | 46243211 |
| 2015 | 6674 | 2497 | 4177 | 5868 | 389 | 139 | 27 | 228 | 47760852 |
| 2016 | 6646 | 2399 | 4247 | 5820 | 383 | 148 | 27 | 247 | 49244195 |
| 2017 | 6784 | 2516 | 4268 | 5932 | 398 | 167 | 25 | 247 | 50858679 |
| 2018 | 6663 | 2449 | 4214 | 5821 | 386 | 159 | 32 | 246 | 52431193 |
| 2019 | 6812 | 2421 | 4391 | 5949 | 387 | 166 | 28 | 271 | 54058263 |
| 2020 | 6991 | 2397 | 4594 | 6082 | 422 | 171 | 30 | 270 | 55659365 |
| Total | 180037 | 66620 | 113417 | 161746 | 9327 | 3140 | 595 | 4874 | 928476665 |

NH, non-Hispanic.

**Supplemental Table 2** Abdominal Aortic Aneurysm-related Mortality, Stratified by Place of Death in older adults in the United States, 1999 to 2020

| **Deaths** | | | | |
| --- | --- | --- | --- | --- |
| **Year** | **Medical**  **Facility** | **Nursing Home/Long-term**  **Care Facility** | **Hospices** | **Home** |
| 1999 | 8655 | 1100 | N/A | 1360 |
| 2000 | 8538 | 1117 | N/A | 1373 |
| 2001 | 7922 | 1168 | N/A | 1330 |
| 2002 | 7737 | 1045 | N/A | 1378 |
| 2003 | 7205 | 1142 | N/A | 1396 |
| 2004 | 6476 | 1060 | 16 | 1423 |
| 2005 | 6290 | 1050 | 54 | 1459 |
| 2006 | 5914 | 1016 | 78 | 1495 |
| 2007 | 5656 | 981 | 103 | 1454 |
| 2008 | 5351 | 914 | 148 | 1405 |
| 2009 | 4921 | 901 | 146 | 1420 |
| 2010 | 4696 | 844 | 182 | 1397 |
| 2011 | 4435 | 809 | 229 | 1461 |
| 2012 | 4124 | 814 | 239 | 1480 |
| 2013 | 4029 | 738 | 250 | 1492 |
| 2014 | 3909 | 722 | 300 | 1516 |
| 2015 | 3793 | 790 | 316 | 1587 |
| 2016 | 3706 | 761 | 354 | 1653 |
| 2017 | 3720 | 813 | 354 | 1687 |
| 2018 | 3568 | 734 | 392 | 1775 |
| 2019 | 3502 | 781 | 411 | 1902 |
| 2020 | 3368 | 748 | 368 | 2244 |
| **Total** | 117515 | 20048 | 3,936 | 33687 |

**Supplemental Table 3** Annual percent change (APC) of Abdominal Aortic Aneurysm–related Age-Adjusted Mortality Rates per 100,000 in older adults in the United States, 1999 to 2020

| Year Interval | APC (95% CI) |
| --- | --- |
| Overall | |
| 1999-2002 | -4.07 (-5.25 to -2.12) |
| 2002-2014 | -5.63 (-6.33 to -5.44) |
| 2014- 2020 | -1.66 (-2.48 to -0.48) |
| Men | |
| 1999-2014 | -6.15 (-6.40 to -5.93) |
| 2014-2020 | -1.38 (-2.33 to -0.18) |
| Women | |
| 1999-2007 | -3.86 (-4.16 to -3.45) |
| 2007-2013 | -5.85 (-7.13 to -5.34) |
| 2013-2020 | -2.78 (-3.36 to -1.93) |
| NH White | |
| 1999-2002 | -3.87 (-4.77 to -2.41) |
| 2002-2013 | -5.60 (-5.94 to -5.42) |
| 2013-2020 | -1.90 (-2.38 to -1.31) |
| NH Black or African American | |
| 1999-2014 | -4.79 (-8.10 to -4.07) |
| 2014-2020 | -2.17 (-4.30 to 4.14) |
| Hispanic or Latino | |
| 1999-2020 | -4.38 (-5.06 to -3.66) |
| NH Asian or Pacific Islander | |
| 1999-2020 | -4.94 (-5.43 to -4.40) |
| NH American Indian or Alaska Native | |
| 1999-2020 | -4.84 (-5.64 to -4.03) |
| Non-metropolitan areas | |
| 1999-2007 | -4.24 (-4.86 to -2.59) |
| 2007-2013 | -6.29 (-9.47 to -5.06) |
| 2013-2020 | -0.94 (-2.01 to 1.30) |
| Metropolitan area | |
| 1999-2001 | -3.48 (-5.39 to -1.96) |
| 2001-2014 | -5.66 (-6.29 to -5.47) |
| 2014-2020 | -1.96 (-2.79 to -0.74) |

APC = Annual percent change; NH = non-Hispanic.

**Supplemental Table 4** Overall and Sex‐Stratified Abdominal Aortic Aneurysm–related Age-Adjusted Mortality Rates per 100,000 in older adults in the United States from 1999 to 2020

| Age-Adjusted Rate (95% CI) | | | |
| --- | --- | --- | --- |
| Year | **Men** | **Women** | **Overall** |
| 1999 | 55.6 (54.3-56.9) | 18.5 (17.9-19.1) | 32.6 (32.0-33.2) |
| 2000 | 54.9 (53.6-56.2) | 18.1 (17.5-18.6) | 32.1 (31.5-32.7) |
| 2001 | 50.3 (49.1-51.5) | 17.6 (17.0-18.1) | 30.1 (29.5-30.6) |
| 2002 | 48.6 (47.4-49.8) | 16.8 (16.3-17.3) | 29.0 (28.5-29.6) |
| 2003 | 46.1 (45.0-47.3) | 16.1 (15.6-16.7) | 27.6 (27.0-28.1) |
| 2004 | 41.5 (40.4-42.6) | 15.0 (14.5-15.6) | 25.2 (24.7-25.8) |
| 2005 | 40.3 (39.2-41.3) | 14.6 (14.1-15.1) | 24.5 (24.0-25.0) |
| 2006 | 37.1 (36.1-38.1) | 14.2 (13.7-14.7) | 23.1 (22.6-23.6) |
| 2007 | 34.5 (33.5-35.4) | 13.8 (13.3-14.3) | 21.9 (21.4-22.4) |
| 2008 | 32.7 (31.8-33.6) | 12.9 (12.4-13.3) | 20.7 (20.3-21.2) |
| 2009 | 30.5 (29.7-31.4) | 12.1 (11.6-12.5) | 19.3 (18.9-19.8) |
| 2010 | 28.5 (27.6-29.3) | 11.4 (11.0-11.9) | 18.2 (17.8-18.6) |
| 2011 | 26.9 (26.1-27.7) | 10.7 (10.3-11.1) | 17.1 (16.7-17.5) |
| 2012 | 24.9 (24.2-25.7) | 10.0 (9.6-10.4) | 16.1 (15.7-16.4) |
| 2013 | 23.6 (22.9-24.4) | 9.6 (9.2-9.9) | 15.2 (14.8-15.6) |
| 2014 | 22.5 (21.8-23.2) | 9.4 (9.0-9.8) | 14.7 (14.3-15.0) |
| 2015 | 22.1 (21.5-22.8) | 9.0 (8.6-9.4) | 14.4 (14.1-14.7) |
| 2016 | 21.9 (21.2-22.6) | 8.5 (8.1-8.8) | 14.0 (13.6-14.3) |
| 2017 | 21.3 (20.7-22.0) | 8.7 (8.4-9.1) | 13.9 (13.6-14.2) |
| 2018 | 20.4 (19.7-21.0) | 8.3 (7.9-8.6) | 13.3 (12.9-13.6) |
| 2019 | 20.6 (20.0-21.2) | 8.0 (7.7-8.3) | 13.3 (12.9-13.6) |
| 2020 | 21.0 (20.3-21.6) | 7.8 (7.4-8.1) | 13.2 (12.9-13.6) |
| Overall | 31.2 (31.0-31.4) | 12.0 (11.9-12.1) | 19.7 (19.6-19.8) |

**Supplemental Table 5** Abdominal Aortic Aneurysm-related Age-Adjusted Mortality Rates per 100,000 stratified by Race in older adults in the United States from 1999 to 2020

| Age-Adjusted Rate (95% CI) | | | | | |
| --- | --- | --- | --- | --- | --- |
| Year | **NH White** | **NH Black or African American** | **NH American Indian or Alaska Native** | **Hispanic or Latino** | **NH Asian or Pacific Islander** |
| 1999 | 35.1 (34.4-35.7) | 19.8 (18.1-21.6) | 26.1 (17.5-37.5) | 13.8 (11.9-15.8) | 19.1 (15.7-22.5) |
| 2000 | 34.5 (33.9-35.2) | 19.8 (18.2-21.5) | 24.3 (16.6-34.3) | 14.5 (12.6-16.5) | 15.7 (12.7-18.7) |
| 2001 | 32.4 (31.7-33.0) | 20.0 (18.3-21.7) | 20.4 (13.0-30.3) | 11.1 (9.4-12.7) | 16.6 (13.6-19.5) |
| 2002 | 31.5 (30.9-32.1) | 16.8 (15.2-18.3) | 23.3 (15.7-33.3) | 11.9 (10.2-13.5) | 15.8 (13.0-18.6) |
| 2003 | 29.8 (29.2-30.4) | 16.1 (14.6-17.6) | 21.4 (14.1-31.1) | 15.2 (13.3-17.0) | 15.7 (13.0-18.3) |
| 2004 | 27.3 (26.7-27.9) | 16.8 (15.3-18.3) | 19.8 (12.8-29.2) | 11.1 (9.5-12.6) | 13.0 (10.7-15.4) |
| 2005 | 26.5 (25.9-27.1) | 16.1 (14.6-17.5) | 18.6 (12.0-27.4) | 11.2 (9.7-12.7) | 15.2 (12.7-17.7) |
| 2006 | 25.0 (24.5-25.6) | 15.9 (14.4-17.4) | 15.3 (9.5-23.3) | 10.6 (9.2-12.1) | 13.8 (11.5-16.1) |
| 2007 | 23.8 (23.2-24.3) | 14.2 (12.8-15.5) | 20.2 (13.7-28.9) | 10.1 (8.7-11.5) | 12.7 (10.6-14.8) |
| 2008 | 22.4 (21.9-22.9) | 13.4 (12.1-14.7) | 20.1 (13.6-28.5) | 10.7 (9.4-12.1) | 11.4 (9.5-13.4) |
| 2009 | 21.0 (20.5-21.5) | 12.3 (11.1-13.5) | 14.5 (8.8-22.4) | 9.0 (7.8-10.3) | 12.4 (10.5-14.4) |
| 2010 | 19.8 (19.3-20.2) | 12.4 (11.1-13.6) | 17.5 (11.7-25.1) | 8.7 (7.5-9.8) | 11.4 (9.5-13.3) |
| 2011 | 18.9 (18.4-19.3) | 11.0 (9.9-12.1) | 13.3 (8.4-20.0) | 7.6 (6.5-8.6) | 9.5 (7.9-11.1) |
| 2012 | 17.6 (17.1-18.0) | 10.8 (9.7-11.9) | 10.9 (6.5-17.0) | 6.9 (5.9-7.8) | 10.3 (8.7-11.9) |
| 2013 | 16.8 (16.3-17.2) | 10.4 (9.3-11.4) | 10.4 (6.4-15.8) | 6.6 (5.6-7.5) | 9.3 (7.8-10.8) |
| 2014 | 16.3 (15.9-16.7) | 9.5 (8.5-10.5) | 12.1 (7.8-17.9) | 6.4 (5.5-7.2) | 8.0 (6.7-9.4) |
| 2015 | 15.9 (15.5-16.3) | 10.1 (9.1-11.2) | 11.7 (7.7-17.2) | 6.6 (5.8-7.5) | 7.3 (6.1-8.6) |
| 2016 | 15.5 (15.1-15.9) | 9.5 (8.6-10.5) | 10.5 (6.8-15.5) | 6.8 (5.9-7.6) | 7.4 (6.2-8.6) |
| 2017 | 15.5 (15.1-15.9) | 9.5 (8.6-10.5) | 9.6 (6.2-14.3) | 6.4 (5.6-7.2) | 7.7 (6.5-8.8) |
| 2018 | 14.8 (14.4-15.2) | 9.0 (8.0-9.9) | 11.0 (7.4-15.7) | 6.1 (5.4-6.9) | 6.9 (5.8-8.0) |
| 2019 | 14.9 (14.5-15.2) | 8.6 (7.7-9.4) | 8.9 (5.8-13.1) | 6.4 (5.7-7.2) | 6.9 (5.8-7.9) |
| 2020 | 14.9 (14.5-15.3) | 8.9 (8.1-9.8) | 10.2 (6.8-14.7) | 6.2 (5.4-6.9) | 6.7 (5.6-7.7) |
| Overall | 21.7 (21.6-21.8) | 12.6 (12.4-12.9) | 14.5 (13.3-15.7) | 8.4 (8.2-8.6) | 10.1 (9.8-10.5) |

NH=non-Hispanic

**Supplemental Table 6** Abdominal Aortic Aneurysm-related Age-Adjusted Mortality Rates per 100,000, Stratified by State in older adults in the United States, 1999 to 2020.

| State | Age-Adjusted Rate (95% CI) |
| --- | --- |
| Alabama | 15.3 (14.7-16.0) |
| Alaska | 19.5 (16.8-22.2) |
| Arizona | 15.1 (14.6-15.7) |
| Arkansas | 21.2 (20.2-22.1) |
| California | 17.5 (17.3-17.8) |
| Colorado | 23.6 (22.7-24.4) |
| Connecticut | 20.4 (19.6-21.2) |
| Delaware | 22.0 (20.2-23.7) |
| District of Columbia | 12.8 (11.1-14.6) |
| Florida | 15.6 (15.3-15.9) |
| Georgia | 13.9 (13.4-14.4) |
| Hawaii | 15.5 (14.4-16.7) |
| Idaho | 22.1 (20.7-23.5) |
| Illinois | 19.4 (18.9-19.8) |
| Indiana | 24.4 (23.7-25.1) |
| Iowa | 24.2 (23.3-25.2) |
| Kansas | 19.0 (18.1-19.9) |
| Kentucky | 22.8 (22.0-23.7) |
| Louisiana | 16.2 (15.5-16.9) |
| Maine | 29.3 (27.7-30.8) |
| Maryland | 19.3 (18.6-20.0) |
| Massachusetts | 20.2 (19.6-20.8) |
| Michigan | 22.0 (21.5-22.6) |
| Minnesota | 28.8 (28.0-29.7) |
| Mississippi | 21.6 (20.6-22.6) |
| Missouri | 21.0 (20.4-21.7) |
| Montana | 22.2 (20.6-23.8) |
| Nebraska | 22.9 (21.7-24.1) |
| Nevada | 16.9 (15.9-17.9) |
| New Hampshire | 23.4 (21.9-24.9) |
| New Jersey | 18.7 (18.2-19.2) |
| New Mexico | 17.9 (16.8-19.0) |
| New York | 16.3 (16.0-16.6) |
| North Carolina | 20.5 (19.9-21.0) |
| North Dakota | 26.0 (24.0-28.1) |
| Ohio | 25.2 (24.7-25.7) |
| Oklahoma | 24.7 (23.8-25.6) |
| Oregon | 23.5 (22.6-24.4) |
| Pennsylvania | 23.7 (23.3-24.2) |
| Rhode Island | 26.3 (24.6-28.0) |
| South Carolina | 19.3 (18.5-20.0) |
| South Dakota | 28.5 (26.5-30.4) |
| Tennessee | 20.7 (20.0-21.4) |
| Texas | 15.9 (15.5-16.2) |
| Utah | 13.0 (12.0-13.9) |
| Vermont | 31.0 (28.5-33.4) |
| Virginia | 17.2 (16.6-17.7) |
| Washington | 22.3 (21.6-23.0) |
| West Virginia | 31.3 (29.9-32.6) |
| Wisconsin | 23.5 (22.8-24.2) |
| Wyoming | 27.9 (25.3-30.6) |
| Overall | 19.7 (19.6-19.8) |

**Supplemental Table 7** Abdominal Aortic Aneurysm–related Age-Adjusted Mortality Rates per 100,000 in older adults in the Metropolitan and Non-metropolitan areas in the United States, 1999 to 2020

| Age-Adjusted Rate (95% CI) | | |
| --- | --- | --- |
| Year | **Metropolitan** | **Nonmetropolitan** |
| 1999 | 31.5 (30.9-32.2) | 37.2 (35.8-38.7) |
| 2000 | 30.9 (30.3-31.6) | 37.1 (35.6-38.5) |
| 2001 | 29.3 (28.6-29.9) | 33.7 (32.3-35.1) |
| 2002 | 28.1 (27.5-28.7) | 33.1 (31.7-34.4) |
| 2003 | 26.4 (25.8-26.9) | 32.9 (31.6-34.3) |
| 2004 | 24.1 (23.5-24.6) | 30.1 (28.8-31.4) |
| 2005 | 23.4 (22.8-23.9) | 29.5 (28.2-30.7) |
| 2006 | 22.2 (21.6-22.7) | 27.5 (26.3-28.8) |
| 2007 | 20.7 (20.2-21.2) | 26.9 (25.7-28.1) |
| 2008 | 19.9 (19.4-20.4) | 24.2 (23.1-25.4) |
| 2009 | 18.4 (17.9-18.8) | 23.7 (22.6-24.9) |
| 2010 | 17.3 (16.8-17.7) | 22.4 (21.3-23.5) |
| 2011 | 16.4 (16.0-16.8) | 20.5 (19.5-21.5) |
| 2012 | 15.3 (14.9-15.7) | 19.5 (18.5-20.5) |
| 2013 | 14.7 (14.3-15.1) | 17.9 (17.0-18.8) |
| 2014 | 14.1 (13.7-14.5) | 17.6 (16.7-18.6) |
| 2015 | 13.7 (13.3-14.0) | 17.8 (16.9-18.8) |
| 2016 | 13.1 (12.8-13.5) | 18.1 (17.1-19.0) |
| 2017 | 13.1 (12.7-13.4) | 17.8 (16.9-18.7) |
| 2018 | 12.6 (12.3-13.0) | 16.7 (15.8-17.6) |
| 2019 | 12.5 (12.2-12.9) | 16.9 (16.0-17.8) |
| 2020 | 12.4 (12.1-12.8) | 17.2 (16.4-18.1) |
| Overall | 18.7 (18.6-18.8) | 23.9 (23.7-24.2) |
